# Materials and prognostic factors of bone regeneration in periapical surgery: A systematic review

**DOI:** 10.4317/medoral.19453

**Published:** 2014-03-08

**Authors:** Alba Sánchez-Torres, Maria Á Sánchez-Garcés, Cosme Gay-Escoda

**Affiliations:** 1 Dentistry Student, School of Dentistry, University of Barcelona, Spain; 2 MD, DDS, PhD, Associate Professor of Oral Surgery. Master’s Degree Program in Oral Surgery and Implantology, School of Dentistry, University of Barcelona, Barcelona. Researcher of the IDIBELL Institute, Barcelona, Spain; 3 MD, DDS, PhD, Chairman and Professor of Oral and Maxillofacial Surgery, School of Dentistry, University of Barcelona. Director of Master’s Degree Program in Oral Surgery and Implantology (EHFRE International University/UCAM/FUCSO).Coordinator/Researcher of the IDIBELL Institute. Head of Oral Surgery, Implantology and Maxillofacial Surgery Department of the Teknon Medical Center, Barcelona, Spain

## Abstract

Objectives: Analyse the effectiveness of different materials and techniques used in guided tissue regeneration (GTR) applied in periapical surgery, comparing the success rate obtained in 4-wall defects and in through-and-through bone lesions as well as to establish prognostic factors. Material and Methods: A Cochrane, PubMed-MEDLINE and Scopus database search (October 2012 to March 2013) was conducted with the search terms “periapical surgery”, “surgical endodontic treatment”, “guided tissue regeneration”, “bone regeneration”, “bone grafts”, “barrier membranes” and “periapical lesions” individually and next, using the Boolean operator “AND”. The inclusion criteria were the use of GTR (bone graft and/or membrane barrier), clinical studies including at least 10 patients, 10 years aged articles published in English or French. The exclusion criteria were case reports and nonhuman studies. Results: 34 publications were selected from a total of 483. 9 of the 34 were excluded. Finally, the systematic review included 25 articles: 2 metaanalysis, 8 reviews, 13 prospective studies and 2 retrospective studies. They were stratified according to their level of scientific evidence using the SORT criteria. The 4-wall periapical and through-and-through lesions improve more their prognosis by combining bone grafts and barrier membranes than using these materials exclusively, respect to the control groups. The results show lower failure rates in 4-wall lesions than in through-and-through lesions using GTR. Conclusions: The combined GTR technique (filling material and membranes) obtains a greater success rate both in 4-wall lesions and in through-and-through lesions, respect to the control groups. The use of regeneration materials seems to be more necessary in through-and-through lesions,> 5mm lesions, lower teeth and apicomarginal lesions as they have the worst healing prognosis. In function of the articles scientific quality, a type B recommendation is given in favour to the use of GTR in association of periapical surgery in case of 4-wall and through-and-through lesions.

** Key words:**Periapical surgery, surgical endodontic treatment, guided tissue regeneration, bone regeneration, bone grafts, barrier membranes.

## Introduction

Endodontic treatment attempts to eliminate bacterial infection in the radicular duct. According to a metaanalysis carried out by Ng *et al*. (1), the probability of success of this treatment ranges from 86% to 93% in a period of 2-10 years following root canal treatment.

Despite a correct endodontic treatment or retreatment, in some cases periapical pathology persists. Therefore, periapical surgery may be indicated considering that is the last therapeutic option previous tooth extraction (2-5). The indications for periapical surgery, based on the protocol proposed by the Spanish Society of Oral Surgery (6-8) are: i) periapical disease affecting a permanent tooth subjected to endodontic treatment (of good quality), with pain and inflammation; ii) periapical pathology with prosthodontic or conservative restoration proven to be difficult to remove; iii) a radiotransparent lesion measuring over 8 to 10 mm in diameter; iv) symptomatic guttapercha overfilling, or presence of a foreign body not amenable to orthograde removal (eg, fractured file); v) other indications (patient requiring endodontic treatment and periapical surgery in a single session, fracture of the apical third, etc.).

These points agree in their majority with those established by the European Society of Endodontology (5), although this one includes one more indication for periapical surgery: perforation of the root or the floor of the pulp chamber.

Guided tissue regeneration (GTR) with the use of barrier membranes and/or bone grafts has been successfully used in different surgical techniques in oral surgery (periodontal surgery, implantology, etc.), just like in periapical surgery to enhance new tissue formation in the defect created by the lesion and by the surgical technique (2,9-16).

The reasons for using regeneration techniques in periapical surgery are to accelerate periapical healing and to allow healing in compromised clinical situations like large periapical lesions (>1 cm), through-and-through lesions (2,5,11,13-15,17-19) and lesions with a periodontal component as apicomarginal lesions (3,5,11,13,14,17,18,20). Although regenerative therapies have great potential, they remain unpredictable in their ability to consistently produce acceptable outcomes in all situations (9).

The aim of the present systematic review is to analyze the materials and results of GTR techniques applied in periapical surgery, comparing the success rates obtained in 4-wall defects and through-and-through lesions as well as to establish prognostic factors.

The focused question regarding to different types of periapical bony defects associated to periapical surgery (4-wall lesions, through-and-through lesions and apicomarginal lesions) is which type/s of biomaterial/s and techniques are the most indicated to achieve a complete osseous regeneration of the defect.

A secondary objective was to establish a list of prognostic factors which can influence in the surgical success.

The available studies in the literature are very heterogeneous, so that it could not be possible to add a statistical study.

## Material and Methods

A Cochrane, PubMed-MEDLINE and Scopus databases search of articles published between October 2012 and March 2013 was conducted. The key words “periapical surgery”, “surgical endodontic treatment”, “guided tissue regeneration”, “bone regeneration”, “bone grafts”, “barrier membranes” and “periapical lesions” were used. Next, the terms were merged using the Boolean operator “AND”, in order to obtain the articles that included two or more of the used search terms.

The inclusion criteria were the use of guided tissue regeneration (use of bone graft and/or membrane barrier) as a part of the surgical protocol, clinical studies including at least 10 patients and 10 years aged articles published in English or French. The exclusion criteria were case reports and non human studies.

The articles selection was agreed by consensus between two of the authors; first by reading of titles and abstracts of the found bibliographic cites to identify the most relevant studies and then, by means of reading the full-text.

## Results

**Figure 1:**
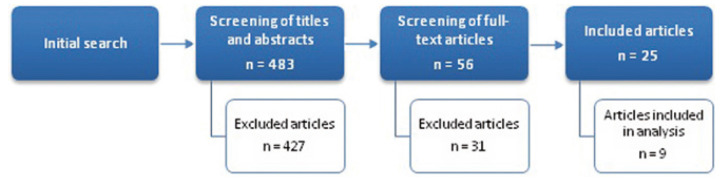
Flow of articles through the systematic review.

Out of the 483 studies obtained initially from the search, the complete text of 56 articles was analyzed by the two authors. 31 of these 56 articles were excluded due to the lack of data and/or lack of direct relationship with the subject and finally, 25 articles with relevance were selected to be included in the systematic review: 2 metaanalysis, 8 systematic reviews, 13 prospective studies and 2 retrospective studies. Specifically, 9 out of the 13 prospective studies have been subjected to the data extraction, synthesis and analysis of the data (11,14-17,20-22) and used to perform a complete analysis about bone regeneration in periapical surgery depending on the technique, the GTR materials and type of lesion (Fig. 1). Eight from nine prospective studies were randomized clinical trials (3,11,15-17,20-22) and only one was a prospective case series (14). The articles were stratified according to their level of evidence, using the SORT (Strength of Recommendation Taxonomy) criteria. Four from nine had a level of scientific evidence of 1 (11,15,17,20) and five, a level 2 (3,14,16,21,22).

A summary which synthesizes the characteristics of each study has been made ( Table 1). The value of average ages from two articles has been calculated by the authors (3,20) because they did not appear in the original text; once the age range had been found, the mean between the two numbers was calculated.

**Table 1:**
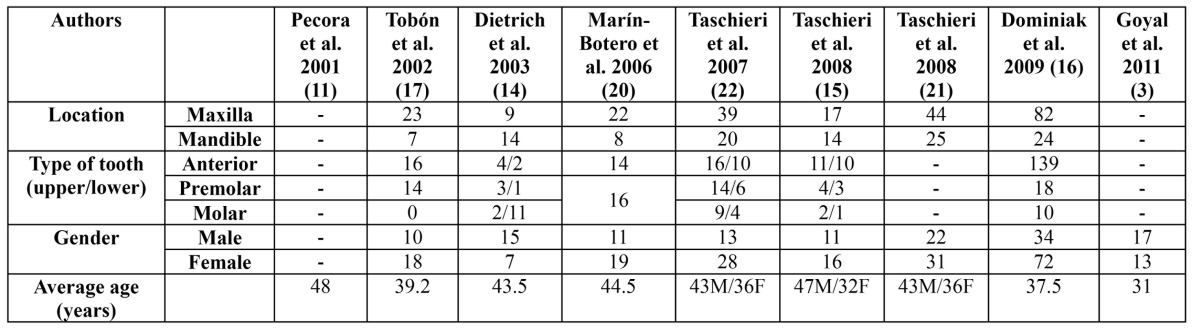
Distribution of teeth, gender and average age of participants in the studies. M: male. F: female.

 Table 2 summarizes the main characteristics of the studies: type of lesion, techniques and criteria assessment; besides, it also includes the number of cases and the percentage of success belonging to each technique, differentiating complete healing from incomplete/uncertain healing or from failure. Not all studies show the value of success in percentage, so some of them have been calculated by the authors (11,15,16,21,22) dividing the number of cases of the determined category (complete success, uncertain healing or failure) between the total cases of the defect type and the used technique, multiplied by 100.

**Table 2:**
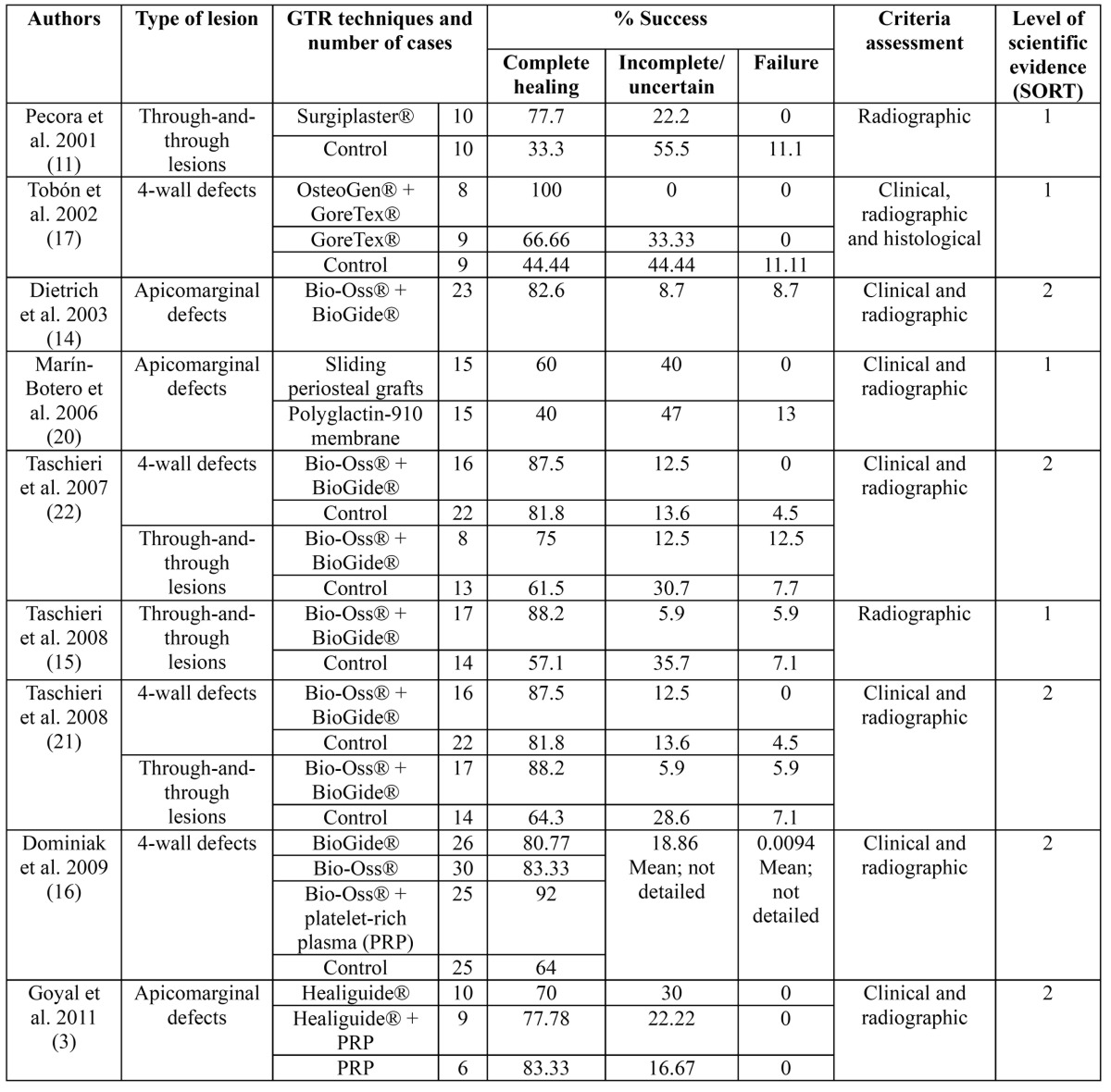
Number of cases, percentage of success at one year follow-up belonging to each technique depending on the case of complete healing, incomplete/uncertain or failure, criteria assessment of the prospective studies and level of scientific evidence (SORT criteria).

The study of methodological quality reveals that from the 9 prospective studies chosen for their analysis only 4 have similarities (16,17,20,21). Of the 5 lasting studies, two are neither randomized nor blinded (14,15), one shows an attrition bias due to loss of participants during the performance of the study (3), another presents a noticeable difference between the number of participants of each group (22) and, concerning to the post-operative evaluation criteria, there are two studies that only use the radiographic criteria (11,15).

The available studies are very heterogeneous, so it has not been possible to carry out a statistical study.

## Discussion

Von Arx and Cochran (18) made a review of the literature to clarify in which type of lesions the use of a membrane is indicated. They proposed a classification of osseous lesions or defects which are distinguished by their location, extension or pathway of infection. Firstly, lesions in class I comprise bony defects located at the apex and are divided in two types: 4-wall or class Ia lesions, that consists of a bone defect confined to periapical region and through-and-through or class Ib lesions, that comprises a periapical bone defect with erosion of buccal and/or palatal/lingual cortical plate. Another type is apicomarginal or endoperiodontal lesions that consist in periapical and concomitant marginal lesions with communication.

This study shows the success rate in the healing of 4-wall lesions with guided regeneration materials varies from 66.66% (17) with the use of a non-resorbable membrane (GoreTex®, W.L. Gore & Associates, Flagstaff, AZ, USA) to 100% (17) combining a synthetic bioactive resorbable hydroxylapatite (OsteoGen®, Impladent Ltd, Holliswood, NY, USA) and a non-resorbable membrane (GoreTex®, W.L. Gore & Associates, Flagstaff, AZ, USA), with clinical, radiographic and histological evaluation. In the control groups, the success rate varies from 44.44% (17), evaluated with clinical, radiographic and histological criteria, to 81.8% (22), with a clinical and radiographic evaluation.

The healing of through-and-through lesions in groups treated with GTR have a success rate that ranges from 75% (22) with the simultaneous use of a xenograft of bovine bone (BioOss®, Geistlich Biomaterials, Wolhusen, Switzerland) and a resorbable bovine collagen membrane (BioGide®, Geistlich Biomaterials, Wolhusen, Switzerland), evaluated clinical and radiographically, to 88.2% (15) with the same combination, evaluated only radiographically. The control groups show a lower percentage of success that varies from 33.3% (11), with a radiographic evaluation, to 64.3% (21) with a clinical and radiographic evaluation.

Regarding the treatment of apicomarginal or endoperiodontal lesions, the success rate is comprised between 40% (20) with the application of a bioabsorbable membrane of polyglactin 910 (Vicryl®, Ethicon, Brunswick, NJ, USA) evaluated clinical and radiographically, and 83.33% (3) with the use of platelet-rich plasma (PRP) and a clinical and radiographic evaluation. There are no control groups for the treatment of these type of lesions.

Periapical surgery provides good access to clean the periapical lesions and the root surfaces and to reshape the surrounding bone and the root apex. The bone close to the lesions is sometimes removed to get better access to the dental apex and healing is almost always by local bony repair (9) which some authors define as the healing of a wound by the new formation of a tissue which does not fully restore the architecture or the function of the preexistent bone (9-12). Regenerative procedures have been introduced with the goal of improving the quality of healing replacing damaged or lost tissue by cells of the same healthy tissue (5,9-12). Complete periapical healing includes regeneration of alveolar bone, periodontal ligament and cementum (2,5,11,12).

The type of healing obtained is critically dependent on the cell type that repopulates the wound first (9,13). Typically, epithelial cells have the fastest migration rate and tend to dominate the initial healing phase. Thus, the exclusion of epithelial cells from the wound allows other cell types with slower regenerative potential to be established (3,9).

The GTR concept was primarily established in periodontal regeneration (3,10,19); even so, it has also been suggested as an adjunct to endodontic surgery (2,3,5,10,11,13,15,17,18,21) and implantology reaching good results, especially in defects with a favourable morphology, self-space maintainers. It represents an effective treatment in terms of healing of periapical lesions, especially in case of through-and-through lesions (2,11,13,15,17,18,21-23).

The most common materials used to obtain regeneration into the surgical field are bone replacement grafts, barrier membranes and host modulating agents like platelet-rich plasma, all considered as tissue engineering (5,9,12,13). Bone grafts are the most used material and they have osteogenic (autograft), osteoinductive (allograft) or osteoconductive (xenograft/alloplast) properties depending on the nature and processing of the graft (10,12).

This systematic review only owns studies effectuated with xenografts and alloplastic grafts. A xenograft refers to tissue taken from one species different from humans, osteoconductive by nature (9,10). Generally, bovine xenograft is used (14-16,21,22). An alloplast is a synthetic or inert osteoconductive foreign body that is implanted into host tissue. Calcium sulphate (5,9,12), hydroxylapatite (17,24), beta-tricalcium phosphate (β-TCP) (24) or calcium phosphosilicate (4,5) are some examples.

Although GTR does not necessarily imply the use of barrier membranes (18), they are the second most commonly used component in these techniques as they prevent apical migration of epithelial cells and connective tissue fibroblasts so that the repopulation of the damaged root surface with periodontal ligament progenitor and stem cells can occur (9,12,15,22). Barrier membranes can be non-resorbable and bio-absorbable. The first are usually expanded polytetrafluoroethylene (e-PTFE) membranes. The need of a second surgery for their removal and its high exposure rate has implied the introduction of other materials as bio-absorbable membranes (5,9) composed of collagen (3,14-16,21,22), polyglactin-910 (20) and other materials such as polylactic acid, polyurethane, acellular dermal matrix, dura mater, chitosan, periosteum and calcium sulphate (9).

Regarding to the treatment for class Ia lesions or 4-wall defects, good long-term results have been obtained with surgical approaches without membrane application. Besides, the authors mention that membranes increase the price of surgical intervention, make the technique more complex and possible can cause complications during the healing period (18). A study made by Dominiak *et al*. (16) shows different treatment options for this type of defects. The greatest percentage of success was obtained with the use of Bio-Oss® combining with PRP (n=25), achieving 92%, while lower percentages of success were obtained with Bio-Oss® (n=30), BioGide® (n=26) and in the control group (n=25) of 83.33%, 80.77% and 64%, respectively.

Conversely, the GTR principle might contribute more favourably in the treatment of lb class lesions called through-and-through lesions (5,18,23). A retrospective study at 4-year follow-up made by Taschieri *et al*. (19) concludes that the use of a membrane in association with endodontic surgery for the treatment of through-and-through lesions leads to excellent outcomes. Pecora *et al*. (11) introduces an alternative by means of using calcium sulphate, a material that may act simultaneously as a filling material and a barrier. The results indicate that it may contribute to improve clinical outcome regarding the control group, although this does not allow us to extract any determinant conclusions.

Autogenous periosteal grafts are an alternative as they act like a barrier membrane. Periosteum can stimulate bone formation when used as a graft because is a structure rich in osteoprogenitor cells. Marín-Botero *et al*. (20) made a study of apicomarginal defects in which a group was treated with sliding periosteal grafts, obtaining a 60% of success and another group was treated with bio-absorbable membranes obtaining a 40% of success. Nonetheless, the authors state that the results have to be considered within the limitations of the study since the sample size is small (n=15 for each group) and in addition, variability of shape and size of the bony defects and the amount of remaining periodontal ligament may influence the regenerative outcome.

Third component of GTR that studies show is platelet-rich plasma (PRP), a host modulating agent. It is a highly concentrated suspension of autologous platelets which secrete bioactive growth factors on activation. They may help to enhance key stages of wound healing and regenerative processes (3,9). Dominiak *et al*. (16) performed a study with 4-wall defects whose participants were divided into four groups: the first group was treated with BioGide®, the second with Bio-Oss®, the third with Bio-Oss® plus PRP and the control group. When compare the second and third groups it was observed that the group treated with Bio-Oss® plus PRP obtained a greater percentage of success (92%) than the group treated with only Bio-Oss® (83.33%); the authors concluded that use of PRP increases the predictability of the regenerative process. Other authors (3) studied the healing of apicomarginal defects and obtained the greatest percentage of success (83.33%) by means of using PRP. Both studies used a clinical and radiographic evaluation.

As is shown in  Table 2, the success rate for GTR in 4-wall defects varies from 66.66% (with the use of GoreTex®) to 100% (combining OsteoGen® and GoreTex®); the success rate for GTR in through-and-through lesions is between 75% and 88.2% (both percentages obtained with the combination of Bio-Oss® and BioGide®). Globally, the percentage of success obtained with GTR in both types of lesions is greater than that obtained in the control groups. GTR has better results in 4-wall lesions than in through-and-through lesions although there is a noticeable increase of outcome and makes the use of regenerative materials more necessary in these cases, considering that these defects often have an awkward healing.

The use of isolated materials such a membrane barrier or a bone graft is not well studied. On the contrary, there is a large number of studies about GTR techniques that combine the two components. The simultaneous use of membranes and bone grafts seems to allow a more predictable healing than isolated techniques. Current literature shows a tendency towards the use of Bio-Oss® and good results are obtained in combining it with BioGide® for the treatment of through-and-through lesions (15,21,22) and apicomarginal defects (14), although the last are considered as the most difficult defects to repair and we have no verified treatment option (5). Regarding 4-wall defects, no failures have been reported for the combined technique that includes the use of Bio-Oss® and BioGide® (21,22) and the combination of OsteoGen® and GoreTex® (17), which denotes that these lesions have a more predictable healing.

Evaluation of periapical surgery results is limited to three modalities: clinical assessment, radiographic evaluation and, in some cases, histological analysis.

Radiographic criteria established for the complete healing and failure groups have been reported to possess a high degree of reliability after 1 year follow-up. Heterogeneity and alterations have been noted in cases assigned to the incomplete healing and uncertain categories. Most studies on periapical surgery use radiographic criteria as the major criterion of success or failure. However, radiographic evaluation is subjected to great variability and observer bias (19,25). Besides, some studies have found that the use of purely xenogenic material resulted in the greater increase in bone density (14,16). This bone substitute is radiopaque and its pattern of resorption and progressive replacement with new bone under different clinical conditions is still a matter of controversy (14,15,19,21). On the one hand, this value may indicate normal bone regeneration in the region analyzed and, on the other, incomplete remodelling of the xenogenic material, resulting in excessive density at the site (16).

Histological analysis of the osseous tissue might be considered as the most reliable technique to assess healing, but it is not routinely performed due to ethical reasons (5,19,23). Peñarrocha *et al*. (25) compared the evaluation criteria from distinct authors used to assess the results of periapical surgery. They consider that Von Arx and Kurt criteria assessment are the most appropriate since they combine as much clinical as radiographic parameters.

Only one study includes a histological evaluation made in the second surgical procedure for non-resorbable membrane removal (17) one year after surgery (n=24). In the control group (n=8), a granuloma was diagnosed in 50% of cases and only 25% showed trabecular bone; cases treated with a membrane barrier (GoreTex®) (n=8) achieved new bone formation in a 62.5%; and in cases treated by means of a combined technique of membrane barrier (GoreTex®) and hydroxylapatite (OsteoGen®) (n=8) exhibited a 100% of new formation of normal trabecular bone.

This systematic review has allowed us to design a list of prognostic factors for GTR in periapical surgery. Several tooth-related factors have been identified to affect the outcome of periapical surgery:

• Amount and location of bone loss (2,3,13-15,18-20,22). The prognosis for smaller lesions after periapical surgery is better than the prognosis for larger ones (6,7,19,22). Delays or alterations in healing have been reported when lesion size was greater than 5 mm (6,19,22).

• Type of defect, lesions classified as Ib or through-and-through lesions have a worse prognosis (22).

• Type of tooth (upper teeth have better outcome) (22).

• Periodontal involvement (14,18).

• Coagulum stabilization thanks to barrier membranes (3).

• Healing criteria used to evaluate the outcome of lesions (25).

Regarding to the investigation implications, more controlled trials are necessary to evaluate bone regeneration in samples with more patients, using barrier membranes and bone grafts in combination or individually and also test with another materials that have different reabsorption rates, to study the treatment of distinct types of lesions.

## Conclusions

GTR is an adjunct technique to periapical surgery that can use a wide variety of techniques and materials. This systematic review allows us to obtain this information about GTR in periapical surgery:

- The combined technique (simultaneous use of bone grafts and barrier membranes) significantly improves the outcome of 4-wall lesions and through-and-through lesions respect to the use of filling materials or barrier membranes alone.

- The data obtained in this study shows that GTR benefits more through-and-through lesions than 4-wall lesions. Through-and-through lesions have a more awkward and unpredictable healing.

- The type of lesions that have a worst prognostic are large lesions (> 5mm), lesions located in lower teeth, defects with periodontal involvement (apicomarginal lesions) and through-and-through lesions. Therefore, GTR constitutes a reliable adjunct technique to periapical surgery to achieve a complete healing in these cases.

- In function of the analysis of the articles and their scientific quality, a type B recommendation is given for use of GTR in 4-wall lesions and through-and-through lesions in order to enhance prognosis.
